# Trends in the Epidemiology of Pandemic and Non-pandemic Strains of *Vibrio parahaemolyticus* Isolated from Diarrheal Patients in Kolkata, India

**DOI:** 10.1371/journal.pntd.0002815

**Published:** 2014-05-01

**Authors:** Gururaja P. Pazhani, Sushanta K. Bhowmik, Santanu Ghosh, Sucharita Guin, Sanjucta Dutta, Krishnan Rajendran, Dhira Rani Saha, Ranjan K. Nandy, Mihir K. Bhattacharya, Asish K. Mukhopadhyay, Thandavarayan Ramamurthy

**Affiliations:** National Institute of Cholera and Enteric Diseases, Kolkata, India; Massachusetts General Hospital, United States of America

## Abstract

A total of 178 strains of *V. parahaemolyticus* isolated from 13,607 acute diarrheal patients admitted in the Infectious Diseases Hospital, Kolkata has been examined for serovar prevalence, antimicrobial susceptibility and genetic traits with reference to virulence, and clonal lineages. Clinical symptoms and stool characteristics of *V. parahaemolyticus* infected patients were analyzed for their specific traits. The frequency of pandemic strains was 68%, as confirmed by group-specific PCR (GS-PCR). However, the prevalence of non-pandemic strains was comparatively low (32%). Serovars O3:K6 (19.7%), O1:K25 (18.5%), O1:KUT (11.2%) were more commonly found and other serovars such as O3:KUT (6.7%), O4:K8 (6.7%), and O2:K3 (4.5%) were newly detected in this region. The virulence gene *tdh* was most frequently detected in GS-PCR positive strains. There was no association between strain features and stool characteristics or clinical outcomes with reference to serovar, pandemic/non-pandemic or virulence profiles. Ampicillin and streptomycin resistance was constant throughout the study period and the MIC of ampicillin among selected strains ranged from 24 to >256 µg/ml. Susceptibility of these strains to ampicillin increased several fold in the presence of carbonyl cyanide-m-chlorophenyldrazone. The newly reported ESBL encoding gene from VPA0477 was found in all the strains, including the susceptible ones for ampicillin. However, none of the strains exhibited the β-lactamase as a phenotypic marker. In the analysis of pulsed-field gel electrophoresis (PFGE), the pandemic strains formed two different clades, with one containing the newly emerged pandemic strains in this region.

## Author Summary

*Vibrio parahaemolyticus* has been associated with several epidemics of foodborne diarrheal infection. Recent observations in several counties have shown the emergence of pandemic strains of *V. parahaemolyticus* with unique genetic features and their role in diarrheal outbreaks. Unlike other enteric pathogens, the appearance of pandemic strains of *V. parahaemolyticus* has not been associated with the economic/hygiene status of the population. The pandemic strains of *V. parahaemolyticus* continue to prevail in Kolkata, India since its appearance during 1996. The present communication describes not only the prevalence of pandemic serovars of *V. parahaemolyticus*, but also the appearance of novel serovars under the pandemic strain category. In addition, the *trh* gene was detected in some of the pandemic strains for the first time. In the newly emerged serovars genetic changes have occurred, as evidenced from the PFGE analysis. Overall, the antimicrobial susceptibility of pandemic strains remains unchanged for the past 20 years. The observations made in this study re-emphasize the importance of this pathogen and shows the recent genetic and serovar changes in the epidemiology of *V. parahaemolyticus*-mediated diarrhea.

## Introduction

*Vibrio parahaemolyticus* is a Gram-negative bacterium, which is normally found in several niches of the coastal environments. In humans, this pathogen causes three major clinical syndromes: gastroenteritis, wound infections and septicemia [1]. Intestinal infections caused by this pathogen are mainly associated with the consumption of raw or undercooked seafood with clinical symptoms such as moderate to severe diarrhea, abdominal cramps, nausea, vomiting, with or without fever and tenesmus [1]. In infected individuals, the frequency of diarrhea may vary from 3 to 10 times per day and in the case of persistent diarrhea; the duration may last for 4–7 days. *V. parahaemolyticus* infection has been reported all over the world, either as sporadic diarrhea or contaminated food-related outbreaks [2], [3]. Generally, the isolation rate of this pathogen from diarrheal cases has been high in Asian countries [4]–[6]. A recent surveillance conducted during 1996–2010 in the US revealed an increase in the infection rate of *V. parahaemolyticus* [7].

To confirm their role in the diarrheal epidemiology, *V. parahaemolyticus* isolated from clinical, food and environmental sources are further tested for virulence and other genetic characteristics. The virulence of this pathogen has been attributed to the production two major factors: thermo-stable direct hemolysin (TDH) encoded by the *tdh*, and TDH-related hemolysin encoded by *trh*. Either or both of these genes have been commonly detected in clinical strains, but not always from food/environmental strains [8]. The emergence of the first pandemic strain of *V. parahaemolyticus* belonging to serovar O3:K6 has been reported from Kolkata during 1996 [9]. Since then, this pathogen has been associated with several large outbreaks of diarrhea in many countries [10].

In addition to virulence characteristics, *V. parahaemolyticus* strains have been tested for the prevalence of different serovars and pandemic marker genes encoded in the ToxRS region by using a group specific PCR (GS-PCR) [11]. This GS-PCR was developed based on the nucleotide sequence variations in the *toxRS* operon, which encode transmembrane proteins involved in the regulation of virulence-associated genes. This specific variation was found only in the pandemic strains of *V. parahaemolyticus* and hence used as a genetic marker for its detection. The *toxRS* gene sequence in the new pandemic strains has difference at 7 base positions compared with non-pandemic strains, of which 2 bases have been used to design primers in the GS-PCR. In an active surveillance of diarrheal infection, we monitor several enteric pathogens among acute diarrheal patients admitted at the Infectious Diseases Hospital (IDH), Kolkata, India. Since multiple antimicrobial resistances have been reported in other enteric pathogens [12]–[15], we examine the susceptibility patterns of *V. parahaemolyticus* strains. In this study, *V. parahaemolyticus* strains isolated during 2001–2012 from the hospitalized acute diarrheal patients were examined for serovar prevalence, virulence traits, antimicrobial resistance and genetic lineage of strains, along with the association of clinical symptoms of the cases.

## Materials and Methods

### Ethics statement

Ethical approval has been obtained from the National Institute of Cholera and Enteric Diseases Ethics Committee (Ref.C-4/2012-T&E), and the enrolled patients/parent in the case of children in this study provided written informed consent.

### Collection of stool specimens

Between January 2001 and December in 2012, every fifth diarrheal patient admitted at the IDH was enrolled in the active surveillance. During enrollment, patients or primary caretakers of children undertook a standardized questionnaire to solicit demographic, epidemiologic, and clinical information. Stool specimens were collected before the administration of antibiotics using sterile catheters and transported to the laboratory with 2 hrs. In the event of any anticipated delay, soaked swabs in stool specimens were stored in Carry Blair transportation medium (Difco, BD, Sparks, MD) at ambient temperature for 6–8 hrs.

### Fecal leucocytes (FLC), red blood cells (RBC) and pH tests

FLC and RBC have been examined microscopically (Olympus CX41, Olympus Corporation, Tokyo, Japan) by smearing a thin layer of fresh stool on a glass slide and counts were made the under high power in five or more fields. Microscopic presence of RBC was further confirmed by Hemaoccult 11 (Smith Kline Diagnostics, San Jose, CA). The stool pH was determined using a portable pH meter (Jenway, Staffordshire, UK).

### Isolation of *V. parahaemolyticus*

Stool specimens/swabs were processed for the detection of *V. parahaemolyticus* after enrichment in alkaline peptone water (Difco) with 1% NaCl and pH 8.5. After 4–6 hrs of incubation at 37°C, a loop full of culture was plated onto thiosulphate citrate bile salts sucrose agar (TCBS, Eiken, Tokyo, Japan), followed by incubation at 37°C overnight. Typical green colonies grown on the TCBS agar have been tested in triple-sugar iron agar, production of cytochrome oxidase, and tolerance to NaCl at various concentrations [16].

### Serotyping

Somatic (O) and capsular antigen (K) of *V. parahaemolyticus* were detected using commercially available kits (Denka Seiken, Tokyo, Japan) that contained 9 pooled polyvalent K group antisera (KI to KIX), 65 monovalent K type antisera (K1 to K71; K2, K14, K16, K27, K35, K62 are not included), and 11 O group antisera (O1 to O11). Freshly grown cultures on nutrient agar (Difco) supplemented with 1% NaCl and heat-killed cells suspended in normal saline were used for K and O serotyping, respectively.

### PCR assays

*V. parahaemolyticus* strains were tested for virulence traits such as *tdh*, *trh* genes and pandemic group specific (GS) *toxRS* gene using PCR assays as described previously [11], [17], [18].

### Antimicrobial susceptibility testing

Antimicrobial susceptibility test was performed by disc diffusion method in accordance with Clinical and Laboratory Standards Institute guidelines [19] using commercially available ampicillin (AM) (10 µg), azithromycin (AZM) (15 µg), ceftriaxone (CRO) (30 µg), chloramphenicol (C) (30 µg), ciprofloxacin (CIP) (5 µg), nalidixic acid (NA) (30 µg), norfloxacin (NOR) (10 µg), ofloxacin (OFX) (5 µg), streptomycin (S) (10 µg), tetracycline (TE) (30 µg), trimethoprim/sulfamethoxazole (SXT) (25 µg), discs (BD, Sparks, MD) in Mueller Hinton agar (MHA) (Difco). These antimicrobials are generally used in the empirical treatment of acute diarrheal cases and hence included in the susceptibility testing. MICs of ampicillin streptomycin and nalidixic acid have been determined by using an E-test (AB bioMèrieux, Solna, Sweden), following the manufacturer's instructions. *Escherichia coli* strain ATCC 25922 was used as the quality control strain for each batch of the assay. Since there is no published interpretive breakpoint to categorize susceptible/resistant *V. parahaemolyticus* strains, we have followed the interpretive breakpoint of *E. coli* strain ATCC 25922 in this study.

### Antimicrobial resistance gene detection

Simplex PCR assays were used to detect antibiotic resistance genes such as *strA*, *aadA1* (encoding aminoglycoside [3′] adenylyltransferases), *bla*_SHV_, *bla_OXA_* and *bla*_TEM_ (encoding β-lactamases) as described before [14], [20]. New primers (VP-bla F-CCTGTTGGTTGGGCTGATGGTT and VP-bla R-GAAGCGAAAGGTCTGTGT CTGTGA) were designed to detect chromosomally encoded *V. parahaemolyticus* beta-lactamase gene (VPA0477) and a *qnr* homologue VPA0095 (QnrVPF- CGAATATCCAGCCCGTCCAGTT and QnrVPR- AATCCAAAGCGCTAGAAGGGTGTA) using a DNA gene sequence of *V. parahaemolyticus* RIMD 2210633 (accession No. BA000032) with the DNAStar software (Madison, WI). Template DNA was prepared by boiling the cultures grown in Luria Bertani (LB, Miller) broth (Difco) for 10 min, rapidly cooled on ice followed by brief centrifugation at 10,000 rpm and the supernatant was used in the PCR.

### Synergy tests

Synergy testing was performed using MHA supplemented with or without the efflux pump inhibitor carbonyl cyanide-m-chlorophenyldrazone (CCCP, 1.5 µM) and ampicillin E-test strips [21].

### Statistical analysis

General log-linear model (GLM) has been used to analyze the association of clinical parameters and stool characteristics with *V. parahaemolyticus* infection. In this analysis, all the variables were treated equally as “response” variables whose mutual association was explored. Using Newton-Raphson with Poisson method, the maximum likelihood parameter estimation model was obtained using SPSS version 19 software [SPSS, Inc., Chicago, IL]. In this analysis, age was grouped in four categories: 1 = up to 10 years, 2 = >10–20 years, 3 = >20–40 years and 4 = >40–≥60 years. The nature of diarrhea was categorized in three groups: 1 = watery, 2 = loose stool and 3 = bloody and mucoid stool. The duration of diarrhea was classified in two groups: 1 = up to 24 hrs and 2 = >24 hrs. Frequency of stool per day was considered in three groups: 1 = up to 5 times, 2 = >5–10 times and 3 = >10 times. Abdominal pain and vomiting were categorized in two groups, each with 1 = present and 2 = absent. Stool characteristics such as the stool consistency, pH, number of RBC, and number of pus cells were made in three categories, each with: 1 = liquid, 2 = mushy and 3 = formed; 1 = <7, 2 = ≥7–8 and 3 = >8; 1 = 1–10, 2 = 11–20 and 3 = absent; 1 = 1–10, 2 = 11–20 and 3 = absent, respectively. The categorical data can highlight the interrelationship in a log linear analysis.

### Pulsed-field gel electrophoresis (PFGE)

PFGE has been made following the PulseNet International protocol [22]. About 40 *V. parahaemolyticus* pandemic strains belonging to diverse serovars have been selected in the PFGE, which includes all the newly identified pandemic serovars (n = 11), representative pandemic serovars (n = 26), along with 3 pandemic O3:K6 strains isolated before 2001 in Kolkata. Briefly, the chromosomal DNA of each strain was digested with *Not*I enzyme (Fermentas, Germany) at 37°C overnight. The *Xba*I (Fermentas) digested DNA of *Salmonella* Braenderup strain H9812 was used as a molecular weight marker. The restriction fragments were resolved in a CHEF Mapper system (Bio-Rad, Hercules, CA). The PFGE patterns were analyzed using the BioNumerics version 4.0 software (Applied Maths, Sint Martens Latem, Belgium) after normalization of the TIFF images with the size standard of strain H9812. Clustering was performed using the unweighted pair group method (UPGMA) and the Dice correlation coefficient with a position tolerance of 1.0%. The PFGE profiles of three O3:K6 pandemic strains isolated before 2001 (VP101, VP174 and VP232 isolated during 1996, 1997 and 1998, respectively) were included in the clonal comparison.

## Results

### Prevalence of serovars

**Figure 1:**
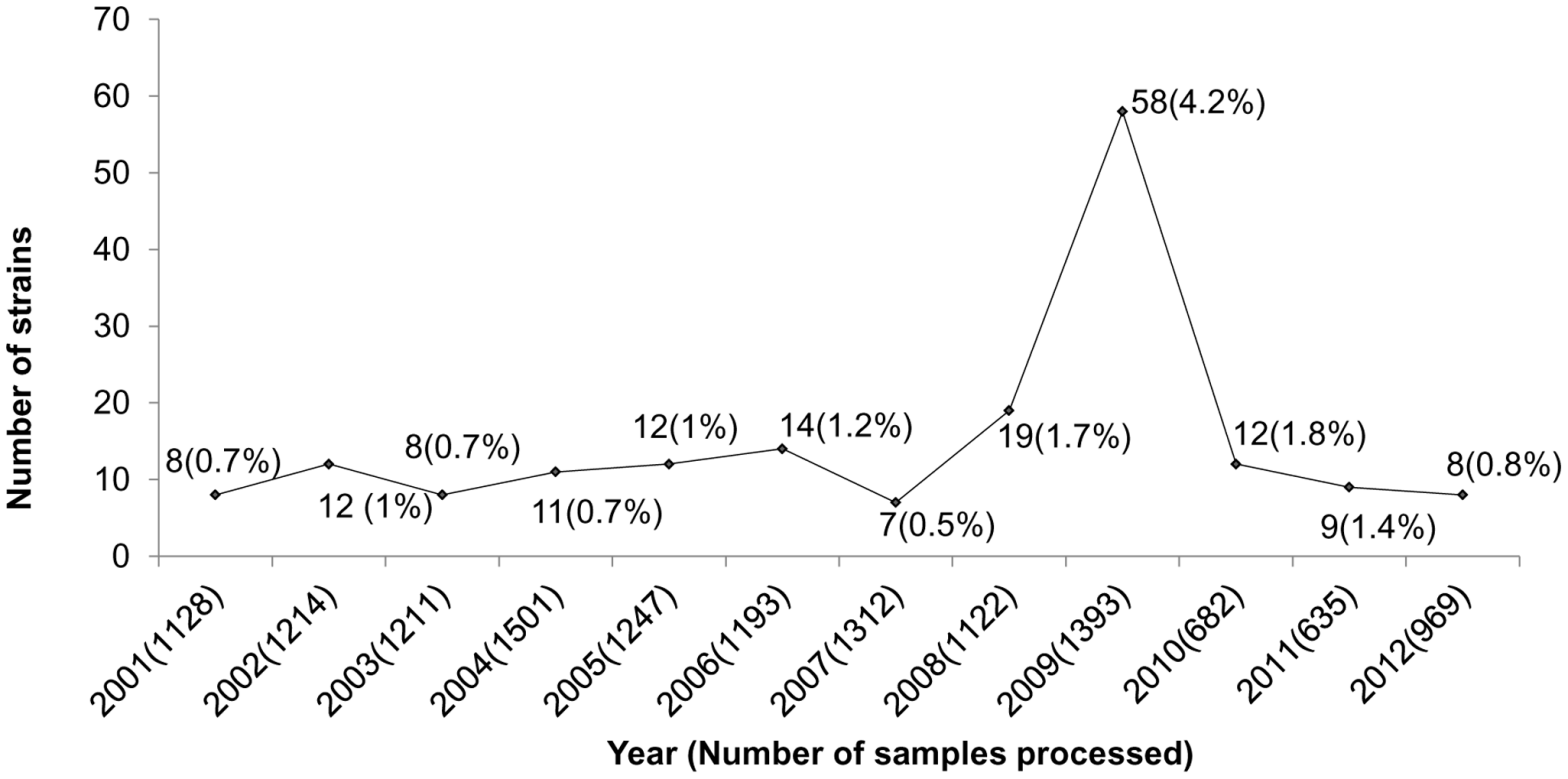
Isolation rate (%) of *V. parahaemolyticus* among hospitalized diarrheal patients in Kolkata.

In a span of 12 years from 2001 to 2012, 178 (1.3%) *V. parahaemolyticus* strains were isolated from 13,607 diarrheal patients. The prevalence of *V. parahaemolyticus* was maximum in 2009 (Fig. 1). Although the isolation rate was low, diverse serovars were identified in this study (Table 1). Overall, the serovars O3:K6 (19.6%), O1:K25 (18.5%), O1: KUT (K-untypable, 11.2%), O3:KUT (6.7%), O4:K8 (6.7%), and O2:K3 (4.5%) were comparatively higher than the others.

**Table 1:** Serovar genotype and antimicrobial resistance profile of *V. parahaemolyticus* isolated from 2001–2012.

Serovar*	*tdh* ^+^ *trh* ^−^	*tdh* ^−^ *trh* ^+^	*tdh* ^+^ *trh* ^+^	*tdh* ^−^ *trh* ^−^	Strain category**		Resistant profile*
					Pandemic	Non-pandemic	
O1:KUT (20)	16	2	1	1	16	4	AM (2); AM,S (18)
O1:K25 (33)	33				33		AM (5); AM,S (25); AM,C,S (1); AM,NA,S (1); # (1)
O1:K30 (2)	1		1		1	1	AM,S (2)
O1:K36 (11)	11				11		AM,S (11)
O1:K38 (3)	2			1	1	2	AM (1), AM,S (2)
O1:K56 (3)	3				1	2	AM,S (3)
O2:K3 (8)	8					8	AM (1), AM,S (7)
O2:K4 (1)	1				1		AM,S
O2:K9 (1)	1					1	AM,S
O3:KUT (12)	11	1			12		S(1), AM,S(11)
O3:K5 (1)	1					1	AM,S
O3:K6 (35)	35				32	3	AM(6); AM,S(25); AM,C,S(1);AM,NA,S(2); # (1)
O3:K30 (1)				1		1	AM,S
O4:KUT (1)	1				1		AM,S
O4:K4 (1)	1				1		AM
O4:K8 (12)	12					12	AM(4); AM,S(7); AM,NA,S(1)
O4:K9 (3)	3					3	AM,S(3)
O4:K11 (4)	4					4	AM,S(4)
O4:K12 (1)	1					1	AM,S
O4:K13 (1)	1				1		AM
O4:K25 (1)	1				1		AM,S
O4:K37 (4)	4					4	AM,S(4)
O4:K55 (2)	2				1	1	AM,S(2)
O4:K68 (3)	3				3		AM,S(2); AM,NA,S(1)
O5:KUT (2)	2					2	AM,S(2)
O5:K15 (1)	1					1	AM,S
O5:K17 (1)	1					1	#
O8:KUT (3)	3					3	AM (1); AM,S(2)
O8:K21 (1)	1				1		AM,S
O10:K60 (4)	4				4		AM(2); AM,S(2)
O10:K61 (1)	1					1	AM,S
OUT:KUT (1)				1		1	AM,C,NA,S,SXT,T
**Total**	**169 (94.9%)**	**3 (1.7%)**	**2 (1.1%)**	**4 (2.2%)**	**121 (68%)**	**57 (32%)**	

* Numbers in parentheses denote number of strains;

**categorized by GS-PCR;

# , susceptible to all antimicrobial agents.

Abbreviations: AM, ampicillin; C, cholarmphenicol; Na, nalidixic acid; S, streptomycin; SXT, trimethoprim/sulfamethoxazole.

### Pandemic and virulence markers

In the GS-PCR, pandemic strains of *V. parahaemolyticus* were detected (68%) more than non-pandemic counterparts (32%). Among the pandemic strain category, serovars O3:K6 (91.4%; 32/35), O3:KUT (100%; 12/12), O1:KUT (80%; 16/20), O1:K25 (100%; 33/33) and O1:K36 (100%; 11/11) were predominantly detected. Though less in numbers, the other new serovars such as O2:K4, O4:KUT, O4:K4, O4:K13, O8:K21, and O10:K60 were identified as pandemic strains in the GS-PCR (Table 1). Based on the virulence gene PCR assay results, *V. parahaemolyticus* strains were categorized in four groups: *tdh* ^+^ *trh* ^+^, *tdh* ^+^ *trh* ^−^, *tdh* ^−^ *trh* ^+^, and *tdh* ^−^ *trh* ^−^. The most predominant virulence gene profile was *tdh* ^+^ *trh* ^−^ (94.9%, 169/178). *V. parahaemolyticus* strains with other gene profiles remained were: *tdh* ^−^ *trh* ^−^ (2.2%, 4/178), *tdh* ^−^ *trh* ^+^ (1.7%, 3/178) and *tdh* ^+^ *trh* ^+^ (1.1%, 2/178). When correlating virulence gene profiles with GS-PCR results, 97.5% (118/121) of the strains harbored only the *tdh* gene. However, 3 *trh* positive strains (2.5%, 3/121) were identified as pandemic strains in the GS-PCR. Of these, two *trh* positive pandemic strains belonged to O1:KUT and the other was identified as O3:KUT. Among the non-pandemic serovars, the *tdh* ^+^ *trh* ^−^ (89.5%, 51/57) profile was predominantly detected. However, 4 (7%) non-pandemic strains did not harbor any of these virulence markers, and 2 (3.5%) had the *tdh* ^+^ *trh* ^+^ profile.

### Antimicrobial susceptibility testing

Ninety-eight percent (174/178) of the strains were resistant to ampicillin, 86% to streptomycin, 3.4% to nalidixic acid, and 1.7% to chloramphenicol. One non-pandemic strain with an unknown serovar (OUT:KUT) was resistant to trimethoprim-sulfamethoxazole, tetracycline, chloramphenicol, nalidixic acid ampicillin and streptomycin. Three strains were found to be susceptible to all the antimicrobials. Ampicillin resistance was common among pandemic and non-pandemic strains. The MIC of ampicillin against 10 randomly selected strains ranged from 24 to >256 µl/ml and 6 to 12 µl/ml for streptomycin. All the strains remained negative for β-lactamase-production.

### Antimicrobial resistance genes

All the strains were screened for *strA*, *aadA1* and *bla_TEM_* genes that encode resistance to aminoglycosides and extended-spectrum β-lactamase (ESBL), respectively. Only two strains harbored *strA*, and one harbored with *aadA1*. All the strains were negative for *bla_TEM_*, *bla_SHV_* and *bla_OXA_* genes. However, the newly reported ESBL encoding open reading frame (ORF) VPA0477 was found in all the strains, including the strains susceptible to ampicillin. Except for two, the chromosomally encoded *qnr* homologue was detected in all the strains, irrespective of the quinolone resistant/susceptible phenotype. The *qnr* homologue negative nalidixic acid susceptible strains had 1–3 folds lower MIC values compared to the strains harboring this gene.

### Involvement of efflux pumps in ampicillin resistance

Synergy test results showed that the MIC of ampicillin was 1.5 to 16-folds less in the selected *V. parahaemolyticus* strains with CCCP as compared to the growth in the inhibitor-free medium (Table 2).

**Table 2:** MICs of ampicillin in presence/absence of CCCP.

Strain ID	Serotype	MIC (µg/ml)	
		AM	CCCP+AM
IDH3704	O10:K60	32	24
J13300	O3:K6	32	16
J10956	O1:K25	32	16
IDH2100	O4:K68	32	16
K12011	O4:K37	24	12
G7140	O4:K68	>256	24
J29017	O3:K5	24	12
L11159	O2:K4	192	12
IDH4492	O1:KUT	32	16
IDH1560	O4:KUT	32	16

Abbreviations: AM, ampicillin; CCCP, carbonyl cyanide-m-chlorophenyldrazone.

### Statistical analysis

The GLM showed a significant association between *V. parahaemolyticus* infection and some of the stool characteristics and clinical symptoms. Liquid and mushy stool consistency, presence of mucus, alkaline stool (pH 8.0), presence of RBC up to 10 and ≥20 FCL counts were significantly associated with the *V. parahaemolyticus* infection (p<0.001) (Table 3). In the older than 30 years age group, short duration of diarrhea (≤24 hrs), frequency of stool more than 5 times/day, the presence of abdominal pain, and high frequency of vomiting were significantly associated with the *V. parahaemolyticus* infection (p<0.001) (Table 4). It is worth to mentioning that in the majority (78.1%; 139/178) of *V. parahaemolyticus* positive cases, this organism was detected as a sole pathogen and in the rest (21.9%; 39/178) as a mixed infection (data not shown). The other pathogens identified in 39 mixed infection cases included *V. cholerae*, *V. fluvialis*, *Salmonella* spp., *Shigella* spp., diarrhegenic *E. coli*, (ETEC, EPEC, EAEC), *Campylobacter* spp., *Aeromonas* spp., Rota virus, Adeno virus, Naro virus, Sappo virus, *Giardia* spp., *Entamoeba histolytica*, and *Cryptosporidium* spp.

**Table 3:** General log-linear model analysis of stool characteristics with *V. parahaemolyticus* infection.

Clinical factor	*V. parahaemolyticus* positive samples n = 122#(%)	Z-values	Estimates (95%CI)	*p*-value
**Consistency**				
Liquid	57(46.7)	4.96	2.64(1.60–3.60)	<0.001*
Mushy	61(50.0)	4.64	2.45(1.42–3.48)	<0.001*
Formed	4(3.3)	reference category		
**Mucus**				
Trace	49(40.2)	−3.87	−0.79(−1.20–−0.39)	<0.001*
Moderate	73(59.8)	reference category		
**pH**				
<7	22(13.0)	−4.55	−1.12(−1.60–−0.64)	<0.001*
≥7–8	2(1.6)	−4.64	−3.34(−4.96–−1.93)	<0.001*
>8	98(80.3)	reference category		
**RBC**				
1–10	83(68.0)	3.59	0.88(0.40–1.37)	<0.001*
11–20	16(13.1)	−1.18	−0.29(−1.04–0.26)	0.239
No count	23(18.9)	reference category		
**Pus cells**				
1–10	54(44.3)	3.71	1.13(0.53–1.73)	<0.001*
11–20	54(44.3)	4.85	1.50(089–2.11)	<0.001*
No count	14(11.5)	reference category		

*Statistically significant;

# , microscopy was carried out only with stool samples (n = 122).

**Table 4:** General log-linear model analysis of clinical factors with *V. parahaemolyticus* infection.

Factor	*V. parahaemolyticus* positive samples n = 178(%)	Z-values	Estimates (95%CI)	*p*-value
**Age**				
Up to 10 yrs	14(7.9)	−3.36	−1.02(−1.62–−0.42)	0.001*
>10–20 yrs	37(20.8)	−0.34	−0.07(−0.52–0.37)	0.734
>20–40 yrs	87(48.9)	4.05	0.77(0.40–1.14)	<0.001*
>40–60 yrs	40(22.5)	reference category		
**Type of diarrhea**				
Watery	144(80.9)	6.81	1.90(1.36–2.44)	<0.001*
Loose	19(10.7)	0.22	0.07(−0.60–0.75)	0.828
Bloody & Mucoid	15(8.4)	reference category		
**Duration of diarrhea**				
Up to 24 hrs	162(91.0)	9.03	3.01(2.35–3.66)	<0.001*
>24 hrs	16(9.0)	reference category		
**Frequency of stool/day**				
Up to 5/day	44(24.7)	1.85	0.41(−0.02–0.84)	0.064
>5–10/day	91(51.1)	5.87	1.45(0.76–1.53)	<0.001*
>10/day	43(24.2)	reference category		
**Abdominal pain**				
Present	101(56.7)	2.27	0.35(0.05–0.65)	0.023*
Absent	77(43.3)	reference category		
**Vomiting**				
Present	148(83.1%)	7.99	1.62(1.22–2.02)	<0.001*
Absent	30(16.9)	reference category		

*Statistically significant.

### PFGE analysis of pandemic strains

**Figure 2:**
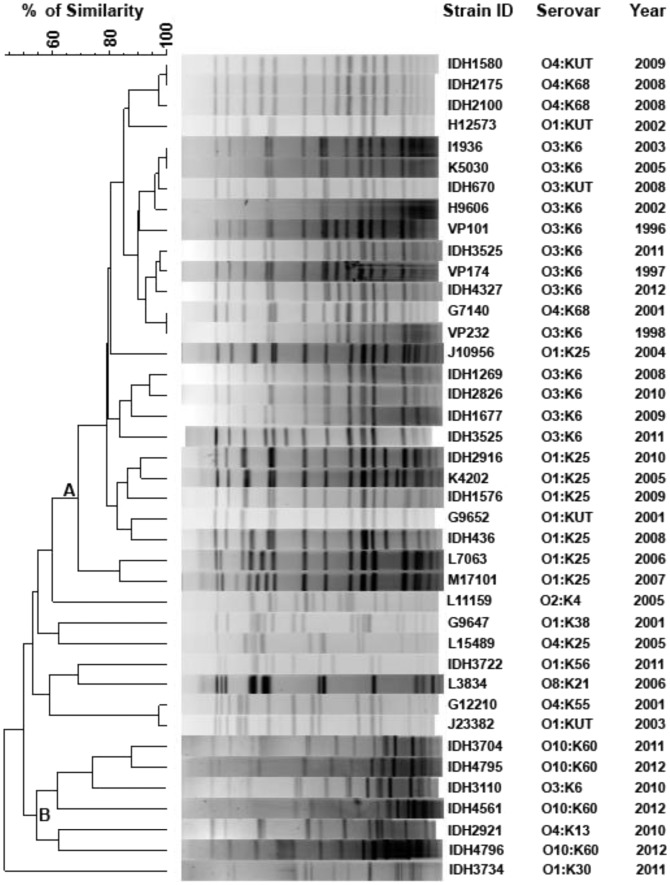
*Not1* digested PFGE profile of *V. parahaemolyticus* with dendrogram. Clustering was performed using the unweighted pair group method (UPGMA) and the Dice correlation coefficient with a position tolerance of 1.0%.

Cluster analysis based on the *Not*I-PFGE profiles revealed two distinct clades (A and B) in the dendrogram (Fig. 2). Clade A had 26 *V. parahaemolyticus* pandemic strains, of which 46% (12/26) of the strains belonged to O3:K6, 27% (7/260) to O1:K25, 11% to O4:K68 (3/26) and 8% to O1:KUT (2/26). All these serovars have been previously reported and had an overall similarity of more than 75%, which includes three O3:K6 strains isolated during 1996–1998. In clade B, the serovar O10:K60 isolated between 2011 and 2012 was more frequent compared to others (57%, 4/7). One unusual O3:K6 serovar was also identified in this clade. From the dendrogram, it appears that the newly emerged pandemic servoars of *V. parahaemolyticus* are heterogeneous with about 50% genetic similarity with serovars placed in clade A (Fig. 2).

## Discussion

Previous studies conducted in Kolkata showed an abrupt appearance of pandemic O3:K6 serovar in 1996 with additional pandemic serovars such as O1:K25, O1:KUT and O4:K68 in subsequent years [9], [23]. Almost during the same period, a similar trend was reported from Thailand and Japan [24], [25]. Spread of pandemic strains of *V. parahaemolyticus* has been reported in several countries, either as a sporadic occurrence or associated with large foodborne outbreaks [10]. In this study, the isolation rate of *V. parahaemolyticus* during 2001–2012 ranged from 0.5% to 4%. The overall isolation rate was 1.3%, which closely matches a report from Bangladesh [26]. In 2009, an increased isolation rate (4.2%) of *V. parahaemolyticus* was detected compared to other years. The rise in the prevalence of *V. parahaemolyticus* during this period was not associated with any local outbreak. In 2009, O1:K36, O1:K25 and O3:K6 serovars were predominantly identified.

Overall, pandemic O3:K6 was isolated throughout the study period. Conversely, only three strains of O4:K68 serovar were identified, which was the second most dominant serovar during 1997–2000 in Kolkata. During 2001–2012, O4:K68 was replaced by serovars O1:K25 (18.5%) and O1:KUT (11.8%). A similar serovar succession has been reported in Thailand [27].

The major *V. parahaemolyticus* pandemic serovars identified in this study were O3:K6, O1:K25, O1:KUT, O3:KUT, O1:K36. Of these, O3:KUT and O1:K36 serovars were newly identified. In addition, O1:K30, O1:K38, O1:K56, O2:K4, O4:KUT, O4:K4, O4:K13, O4:K25, O4:K55 and O8:K21 and O10:K60 serovars were also positive in the GS-PCR assay and hence considered pandemic strains. Studies conducted in Peru, Norway and Chile have also shown emergence of new GS-PCR positive serovars such as O3:KUT, O3:K58, O3:K68 [28]–[30]. Universally, all the pandemic strains have 7 base variations in the nucleotide sequence of *toxRS* operon, which encodes transmembrane proteins involved in regulation of virulence-associated genes. These distinctive gene mutations were found in the non-pandemic strains of *V. parahaemolyticus*. Based on our results and other reports, it appears that several new serovars have emerged recently with pandemic strain attributes. However, in southern Thailand, the major pandemic serovars remained consistent for more than 6 years [24]. The other noteworthy aspect of this study was the emergence of *trh*-harboring pandemic strains. Generally, pandemic strains of *V. parahaemolyticus* harbor only the *tdh* gene. The *trh* gene association has not been reported previously. Serovars O1:KUT and O3:KUT harbored the *trh* gene, and the other two *tdh* and *trh* positive strains belonging to O1:KUT and O1:K30 were negative in the GS-PCR.

Several investigations have shown that clinical strains of *V. parahaemolyticus* are susceptible to many antimicrobial agents as compared to environmental strains [27], [31], [32]. Recently, ESBL-production and fluoroquinolone resistance was reported in *V. parahaemolyticus* isolated from food samples [32], [33]. *V. parahaemolyticus* remained highly susceptible to many antimicrobial agents, despite the fact that other enteric pathogens have developed multiple antimicrobial resistances in this region [12]–[15]. In other countries, ampicillin/trimethoprim-sulfamethoxazole resistance has been reported in *V. parahaemolyticus* [24], [27], [34].

It is known that ampicillin resistance is very common in *V. parahaemolyticus* [9]. Following this trend, 98% of the *V. parahaemolyticus* strains isolated in the present study showed resistance to ampicillin. However, in the MIC assay, ampicillin resistance varied from moderate to high level, with selected strains belonging to different serovars. When examined for the mechanism of ampicillin resistance, we found that the resistance was not related to the tested *bla* gene alleles, as all the strains were negative in the PCR assays. Ampicillin resistance was also not related to a chromosomally encoded β-lactamase ORF (VPA0477; accession no. BA000032) as this encoding gene was detected in both susceptible and resistant strains. In *V. parahaemolyticus*, the beta-lactamase ORF (VPA0477; accession no. BA000032) has not been annotated consistently in pandemic and pre-pandemic strains of genomes sequenced (accession nos. BA000032 and CP003973) and hence there is no experimental proof for the functional aspect of this encoding gene. However, we found that the observed ampicillin resistance was mediated by an efflux system. This mechanism was demonstrated by synergistic testing with the efflux pump inhibitor CCCP. The MIC of ampicillin for *V. parahaemolyticus* decreased considerably when tested with CCCP at the highest concentration 1.5 µM. When the concentration of CCCP increased to 2 µM and above, the growth of *V. parahaemolyticus* was inhibited.

Streptomycin was the other antimicrobial agent for which most of the *V. parahaemolyticus* strains were resistant. The MIC of streptomycin revealed that resistance was close to that of the susceptibility cutoff value (>8 µg/ml) in *E. coli* [35]. The mechanism of resistance for this antibiotic in *V. parahaemolyticus* was not related to the presence of *strA* or *aadA1*, as these genes were found in only three strains.

The pandemic and non-pandemic strains were susceptible to trimethoprim-sulfamethoxazole, ceftriaxone, fluoroquinolones, and very few pandemic strains were resistant to chloramphenicol and nalidixic acid. The chromosomally encoded *qnr* homologue VPA0095 (accession no. BA000032) have more than 50% similarity with the plasmid-mediated *qnrA* and *qnrS* [36]. This *qnr* homologue was detected in 176 of 178 strains screened in this study. Although these two strains displayed susceptibility for fluoroquinolones, the MIC value for nalidixic acid was 1–3 fold less compared to strains that harbored the *qnr* homologue VPA0095.

*V. parahaemolyticus* infection has been significantly associated with older age group with clinical symptoms of abdominal pain, nausea, vomiting and bloody stool [5]. We found that stool specimens of *V. parahaemolyticus* infected cases were significantly detected with alkaline pH with high RBC and FLC counts. A high RBC and FLC count in the stool is an indication of an inflammatory diarrhea. In healthy individuals, the pH progressively rises in the small intestine from pH 6 to 7.4 in the terminal ileum. The pH falls to 5.7 in the caecum and steadily increases to pH 6.7 in the rectum [37]. Due to large secretion of small intestinal fluid, the pH of diarrheal stool remains alkaline when excreted. The alkaline pH favors many enteric vibrios and considerably reduces the normal gut flora [38]. We are not ruling out the possibility of involvement of other pathogens as mixed infections among diarrheal patients. It is worth mentioning that in the majority of the *V. parahaemolyticus* positive cases, this organism was detected as a sole pathogen indicating the importance of *V. parahaemolyticus* as one of the major etiological agents of diarrhea in this region.

Previous reports revealed clustering of *V. parahaemolyticus* O3:K6 and O4:K68 serovars from India and Thailand with 78–91% similarity in the PFGE profiling [39]. In the subsequent years, several other serovars were genetically grouped with O3:K6 [27], [40]. In this study, we found that pandemic serovars such as O3:K6, O1:K25, O4:K68 and O1:KUT were clustered in one clade and several new serovars remained in the other. The overall similarity between the old pandemic serovars with new serovars remained only about 50%. Recently, similar genetic event has not been reported among pandemic strains of *V. parahaemolyticus*.

### Conclusion

In this surveillance study, we found variation in the isolation rates of *V. parahaemolyticus* from hospitalized acute diarrheal patients. Combined genetic and molecular typing analysis verified emergence of newer pandemic serovars in this region. The tested *V. parahaemolyticus* strains reveled susceptibility towards a wide range of antimicrobials used in the treatment of diarrheal infection.
